# Learning how relationships work: a thematic analysis of young people and relationship professionals’ perspectives on relationships and relationship education

**DOI:** 10.1186/s12889-022-14802-5

**Published:** 2022-12-13

**Authors:** Simon Benham-Clarke, Jan Ewing, Anne Barlow, Tamsin Newlove-Delgado

**Affiliations:** 1 School of Education, University of Exeter, Exeter, UK; 2 School of Medicine and Health, University of Exeter, St Luke’s Campus, Room 2.05, South Cloisters, Exeter, EX1 2LU UK; 3 Law School, University of Exeter, Exeter, UK

**Keywords:** Relationship, Education, Curriculum, Romantic, Teachers, School, Mental health, Well-being, Public health, Young people

## Abstract

**Background:**

Relationships in various forms are an important source of meaning in people’s lives that can benefit their health, wellbeing and happiness. Relationship distress is associated with public health problems such as alcohol misuse, obesity, poor mental health, and child poverty, whilst safe, stable, and nurturing relationships are potential protective factors. Despite increased emphasis on Relationship Education in schools, little is known about the views of relationship professionals on relationship education specifically, and how this contrasts with the views of young people (YP). This Wellcome Centre for the Cultures and Environments of Health funded Beacon project seeks to fill this gap by exploring their perspectives and inform the future development of relationship education.

**Methods:**

We conducted focus groups with YP (*n* = 4) and interviews with relationship professionals (*n* = 10). The data was then thematically analysed.

**Results:**

Themes from YP focus groups included: ‘Good and bad relationships’; ‘Learning about relationships’; ‘the role of schools’ and ‘Beyond Relationship Education’. Themes from interviews with relationship professionals included: ‘essential qualities of healthy relationships’; ‘how YP learn to relate’ and ‘the role of Relationship Education in schools’.

**Conclusions:**

YP and relationship professionals recognised the importance of building YP’s relational capability in schools with a healthy relationship with oneself at its foundation. Relationship professionals emphasised the need for a developmental approach, stressing the need for flexibility, adaptability, commitment and resilience to maintain relationships over the life course. YP often presented dichotomous views, such as relationships being either good or bad relationships, and perceived a link between relationships and mental health. Although not the focus of current curriculum guidance, managing relationship breakdowns and relationship transitions through the life course were viewed as important with an emphasis on building relational skills. This research suggests that schools need improved Relationship Education support, including specialist expertise and resources, and guidance on signposting YP to external sources of help. There is also potential for positive relationship behaviours being modelled and integrated throughout curriculums and reflected in a school’s ethos. Future research should explore co-development, evaluation and implementation of Relationship Education programmes with a range of stakeholders.

## Background

Relationships in various forms are an important source of meaning in people’s lives that can benefit their health, well-being and happiness [1]. ‘A ‘distressed’ relationship is one with a severe level of relationship problems, which has a clinically significant negative impact on their partner’s wellbeing. Those in ‘distressed’ relationships report regularly considering separation/divorce, quarrelling, regretting being in their relationship, being unhappy in their relationship, for example’ [2]. A growing evidence base shows that distress in relationships is associated with public health priorities such as alcohol misuse, obesity, mental health problems, and child poverty, whilst safe, stable, and nurturing relationships are potential protective factors [3–5]. For young people (YP), there is evidence of a significant link between well-being and romantic relationships, suggesting that these relationships (when healthy) can positively influence self-concept, social integration and social support [6]. However, research indicates that some early romantic relationships can act as stressors regardless of their nature, whilst YP are negotiating other developmental tasks. For example, Olson and Crosnow’s longitudinal analysis [7] suggested that adolescent romantic relationships are associated with increased depressive symptomatology, particularly for girls.

The term ‘relationship’ has been defined as an enduring association between two persons [8]. The terms ‘healthy’ or ‘quality’ relationships have been described, defined and measured in various ways. They are ‘complex and ambiguous constructs’ with factors varying for each type of relationship [9]. Attempts to reach a definition tend to focus on interaction and positive and negative relationship characteristics and behaviours such as the existence or absence of caregiving, respect, support, emotional regulation, and the ability to learn from experience [10, 11]. It has been theorised that early intervention and the development of these relationship skills in YP may allow them to negotiate early romantic relationships better as well as improve the quality and/or health of adult relationships, normalise help-seeking behaviour and prevent or manage relationship breakdown [12, 13]. In their 2014 Manifesto, the Relationships Alliance^1^ called upon The Department for Education (DfE) “to develop standards for those delivering RSE (Relationship and Sex Education) and set an expectation that schools recognise that developing relational capability is an important function of education and a child’s future” [14]. Relational capability refers to the capacity to form and maintain safe, stable, and nurturing relationships [15].

In 2019, DfE published statutory guidance in England on Relationship and Sex Education (RSE) [16], following the passing of the Children and Social Work Act 2017 [17]. The new Act stipulates^2^ that pupils should learn about safety in forming and maintaining relationships; the characteristics of ‘healthy’ relationships and how relationships may affect physical and mental health and well-being. However, schools have been largely left to work out how to deliver this sensitive area of education, with little practical content guidance to date [18]. Skills for ‘healthy’ romantic relationships have also been relatively neglected both in research and practice. There are several programmes developed for YP that teach about relationships, but those that currently exist are mainly from the US, and generally focussed on sexual health or relationship violence [19, 20]. Similarly, research with YP on their perspectives of RSE mostly focus on their views on sex education [21]. Therefore, despite the increased emphasis on delivering RSE in schools,^3^ little is known about how YP view this aspect of the curriculum, or what outcomes they feel it should deliver. This is an important gap to fill to engage YP with the curriculum, and to lay the groundwork for the design, adaptation and evaluation of healthy relationship programmes. Patient and Public Involvement (PPI) work conducted in a prior project [22] by some of the authors demonstrated a great appetite in YP to learn more about relationships.

Our Beacon project, funded by The Wellcome Centre for the Cultures and Environments of Health, is focussed on ‘Transforming relationships and relationship transitions with and for the next generation’ in two strands (Healthy Relationship Education (HeaRE) and Healthy Relationship Transitions (HeaRT)). As part of the project, we conducted qualitative interviews and focus groups with young people and relationship professionals, with the aims of exploring their perspectives on relationships and relationship education. This paper presents and integrates the findings of these studies, to inform the development of future Relationship Education.

## Methods

### Recruitment

YP were recruited from a convenience sample of community groups and schools in South-West England, across urban, suburban and rural settings. Young people were contacted through school and youth group leaders, who made the first approach to participants. YP consented for themselves if aged 16 and over; for under 16 s, both parent and young person consent was sought. The YP formed four focus groups with a total of 24 participants. The two focus groups conducted in schools were with Years 9 and 10 pupils (aged 14 to 16 years). Following PPI consultation, these were set up separately for boys and girls; one group with eight girls and one with seven boys. The community group focus groups included young people aged between 14 and 18 and had one group with four boys and one with two boys and three girls.

A purposive sampling strategy was used to recruit the relationship professionals, seeking out key people who are likely to provide rich sources of information or data [22]. Here, ten nationally based relationship professionals (three men and seven women) were purposively sampled for their recognised expertise in the field of romantic relationships either through their research interests or because they were psychotherapists or counsellors. All had a minimum of 15 years of experience in their chosen field, and most had many more. Consent in writing or by audio recording was obtained before the interview.

### Procedure

Focus groups with YP were used due to their suitability for exploring ideas within their social context [23, 24]. The topic guides were developed and refined through accompanying consultations with YP in our Youth Panel PPI sessions. Content included questions and prompts around views on relationships, experiences of Relationship Education, and what YP wanted to get from participating in Relationship Education. The first two focus groups were conducted face-to-face in February 2020. Due to COVID-19, the procedure had to be adapted for the latter two, which were conducted on Microsoft Teams in the summer of 2020. The focus groups were audio-recorded and conducted by TND and SBC with each lasting approximately an hour.

Semi-structured telephone interviews were conducted with the relationship professionals by JE. An interview schedule for the relationship professionals was devised, piloted and refined in team discussions. The topics relevant to this paper were the views of the relationship professionals on what constituted an enduring, mutually satisfying intimate partner relationship, how older children can learn the skills needed to identify healthy and unhealthy relationships and the role, content and delivery of Relationship Education. The interviews were conducted by telephone since there are no significant differences between telephone and face-to-face interview data [25] and given COVID-19 restrictions at the time. The duration of each interview was 64 min on average.

### Analysis

The focus groups with YP and the interviews with professionals were analysed separately rather than in combination, as interview schedules and formats were different for both. Transcription was conducted by an approved University service. NVivo 12 was used to manage the data, analysed using the thematic approach described by Braun and Clark [26]. In both datasets, a second author coded the first transcripts. Variations between coders were discussed by the team. Themes were developed separately for the YP and the relationship professionals; in this paper we present and compare these themes, identifying difference and similarities in the Discussion section.

### Ethical approval

Ethical approval was gained from the University of Exeter Medicine School (UEMS) Research Ethics Committee (reference: Jun20/D/229∆1) for the research with YP and the University of Exeter College of Social Sciences and International Studies Research Ethics Committee for research involving relationship professionals (reference: 201,920–017).

The ethical approach we took is based on the successful and tested approach used by the Shackleton Project (UEMS ethics number 201617–018). We developed a protocol, agreed with teachers and community group leaders, for actions to be taken should a participant appear distressed, wish to withdraw, or should concerns be raised. We were highly aware that this could be a sensitive area, and emphasised to participants that they could withdraw at any point, as well as ensuring that they were aware of sources of support, and of confidential ways to contact the researchers, teachers, or community group leaders (e.g. through private chat on Teams) if they needed to. Researchers were alert throughout the groups for verbal and non-verbal signs that YP might wish to leave or take a break from the discussions, and strategic pauses or break points were included to facilitate this. The researchers were both experienced and well placed to conduct the focus groups with YP. The topics discussed with YP were framed to young people as being around ‘healthy relationships’ and existing RSE guidance. Our approach throughout the research was to engage young people in helping us to understand how Relationship Education could be improved for all YP in general. We used and explained Chatham House Rules to participants but were aware that this is not sufficient as the only measure. Therefore, we used appropriate distancing techniques, discouraging and steering conversations away from personal disclosures as needed and framing questions accordingly, for example, ‘what should young people get out of Relationship Education? We developed a protocol, agreed with teachers and community group leaders, for actions to be taken should a participant appear distressed, wish to withdraw, or should concerns be raised.

All names referred to below are pseudonyms.

## Results

### Young people

#### ‘Good’ and ‘bad’ relationships

When asking what was meant by relationships YP appeared to be most comfortable and forthcoming in discussing relationships using dichotomous terms. Typically, relationships were categorised as positive or negative, such as good, bad, right, wrong, comfortable, uncomfortable, successful, unsuccessful, healthy and unhealthy. There was also a frequently expressed concept of ‘normal’ versus ‘abnormal’ relationships, which linked to a desire to be taught how to have a ‘normal relationship’, although few participants challenged this.

*’Like there are bad sides of a relationship, there’s the good side of the relationship’.* (Male)

*’I don’t think I was ever taught in school about what a normal relationship is or how a relationship works’.* (Male)

*‘… I don’t want to be too forceful in this cookie-cutter idea of what good and bad relationships are, ... people are free to do what they want’.* (Female)

YP attempted to define the qualities involved in ‘healthy’ or ‘normal’ relationships differently. Trust, respect and having common ground were often mentioned. Communication was also seen as being crucial, which linked to handling conflict the ‘right’ way. They also recognised that these qualities were involved in the different stages of relationships.

*‘Well, I think a lot about healthy relationships in general is to do with communication. And starting a relationship and establishing what you want from the relationship is very important, and the same with finishing a relationship and saying to someone “I’m not happy with this because of this, this and this … . So, I think all of those stages really are about communication’.* (Female)

Some YP introduced different sources of influence on relationships. They attributed importance to the role of upbringing and parental models. Again, ‘normality’ in relationships was present as a concept.

*‘I think our parents are our closest role models really’.* (Male)

‘*if you’ve been brought up in a domestic violence place or household, you’re never going to know until you grow up “Oh, that’s not okay, that’s not a normal thing*’. (Male)

In response to a question about how Relationship Education might help young people in different stages of their lives others commented on the influence of fairy tales, and Disney in particular; this was linked most strongly to gender roles and expectations in relationships.

*‘I think it actually does create this toxic image to some degree… it’s very much the female is feeble, and she must be saved by the male, and it kind of creates a toxic masculinity’.* (Female)

*’It’s embedded into our heads that it’s always Prince Charming and it’s always the prince and the princess … you don’t understand it until you actually get to it, and that’s when you realise that it’s not like Disney movies or anything ...’.* (Female).

Participants recognised that these ‘bad’ relationships early in life could have long-lasting impacts, including on mental health. This extended to the relationships between parents and children.

*‘I’ve got so many friends who have fallen down mental health spirals due to bad relationships’.* (Female)

*“Some parents, because they had such a rough childhood, treat their children the same thinking that it is the right way’.* (Female).

#### Learning about relationships

There was a general feeling from many participants that Relationship Education would have a range of benefits for YP, across different kinds of relationships. Communication and conflict were critical areas where participants felt that there were skills, or ways of coping or doing things that they could learn.

*’how to communicate effectively with our peers and partners, family members’.* (Female)

’*[I would like to learn] Probably how to defuse an argument, … instead of having to shout at each other and maybe possibly break stuff and maybe even harm each other, and you can talk about it responsibly’.* (Male)

Some of the desired outcomes involved learning how to manage different stages in relationships; for example, how to sustain happy relationships, and how to end relationships that could not be sustained, and cope with the aftermath. There was also a sense that they were sometimes taught about ‘red flags’ (signs that relationships are unhealthy), but not how then to end the relationships.

’*the basic foundations of relationships, like how to keep it running, happy…’.* (Male)

*’if you’ve tried to maintain them but it… keeps happening, you just need to know how to end it nicely’.* (Male)

*’It is all well knowing the signs, but if you don’t know how to get out of an unhealthy relationship what is the point of knowing that it is unhealthy?’* (Female)

Some participants felt that focussing on relationships with themselves as a first step would have greater long-term benefits and could help YP avoid abusive relationships. One participant had their own experience of where they felt Relationship Education had an impact on their well-being but thought it would have been more beneficial if taught sooner.

*’… that is a big thing for people our age more – accepting themselves rather than being in a relationship with other people. Their mental health more than other people’.* (Female).

’*it has made me be more… conscious of my relationships and friendships, and I’m able to see which ones are bad and been able to cut off bad relationships…my mental health would be better now if that education had happened earlier’.* (Female)

Some YP were thinking about how relationships might be challenged after leaving school or relocating, and how Relationship Education might prepare them for that, whilst others thought further ahead to when they might have families, and the potential impacts of Relationship Education in the longer term.

*‘[after relationship education] If they were a parent, they would know how to treat their children and instead of the way their parents treated them, treat them a different way’.* (Female)

#### The role of schools

YP saw schools as offering a neutral setting in which Relationship Education can be taught free from the potential influences and biases. This was thought to be critical, particularly for those who might have more challenging backgrounds, however a desire was expressed for a greater focus in schools on how relationships ‘work’ rather than on sex education.

*‘people need to be taught about relationships in quite an unbiased environment, and school is the most likely place that’s going to happen’.* (Female)

*‘[Relationship lessons have] been very clinical. It’s not really teaching you anything to do with how the relationship works … For me, it’s just been the clinical side of sex, basically’.* (Male)

In terms of *how* Relationship Education should be taught, YP emphasised the need to build on earlier learning and to revisit important content. Participants also felt that talking about family and peer relationships should come first, building up to later discussions about romantic relationships in later years at school, with some highlighting links between patterns of relationship behaviour.

*’ I think they need to talk about our family relationships before they talk about future ones that we will have’.* (Female)

*’even in primary school, you have friendships and stuff. And if you’re getting bullied, you might not necessarily realise the way that they’re using you and being mean to you. And if you get used to that from a young age … it’s very hard to get out of that pattern of ending up with people who aren’t necessarily a good influence on you’.* (Male)

Some YP were concerned about whether education about romantic relationships could put YP under pressure if it were too early, but others felt this could leave young people open to abuse.

*‘… you can’t teach them too much at a young age, otherwise they’ll feel like there’s a lot of pressure on them when it comes to relationships’.* (Male)

*‘the younger ages are the most susceptible to abuse… because you don’t have the knowledge’.* (Female)*.*

#### Beyond relationship education lessons

Discussions about teaching in school led to several YP voicing reservations about the limits of what Relationship Education could do, and acknowledgements of the complexity of whether relationships can be ‘taught’ at all.

*‘I think first and foremost, it’s the role of the parents to teach about relationships. And I think all the school can really do is build on that’.* (Male)

*‘…to teach it, the first thing that you need to do as a teacher would be acknowledge that it isn’t necessarily something that can just be taught, and it’s more complicated than that*’. (Female)

There was a feeling amongst participants that schools could improve relationship outcomes for YP in other ways beyond the Relationship Education lesson, such as having someone to talk to, in person and in private. Others wanted signposting and information about sources of help outside the school setting.

*‘I think it’s important, especially with young people, to have someone to speak to…Maybe a counsellor or something’.* (Female)

*‘it needs to offer information of places where people who might be in unhealthy relationships can go’.* (Female)

YP felt help was needed beyond RSE especially when a relationship was ending, in terms of specialist and peer support, and some even made the case for access to ‘experts’ for relationship breakdown related problems.

*‘it’s hard to teach people about how to deal with a break-up…But that’s why I think people who are experts on relationships should probably be better at it’.* (Male)

### Results- relationship professionals

#### Theme 1 – qualities of healthy relationships

The quality of a healthy relationship most frequently cited by the relationship professionals was communicating well. As Rosemary Allen put it.

*’the ability to be able to express what you think, what you need and to be able to hear the other person… being able to… adapt language so that you are using the tone and the quality and the vocabulary that gets across what you need to say and being sure that it is understood’.*

Secondly, an ability to adapt was thought to be critical, and this required the couple to be flexible – sufficiently flexible to learn from one another but also to adapt to life’s transitions such as having a baby or children leaving home, which Alexander Ingles said depended on a.

‘*flexibility of internal world. It's about whether one is potentially available for development throughout life’.*

The skills needed to adapt can be learned, and some potentially ‘unpromising’ relationships can flourish provided one person in the couple is sufficiently skilled and flexible at the outset. The relationship professionals agreed that couples who have the degree of flexibility required, such that their relationships thrive over time, tend to be ‘developmental’ in outlook that is, they expect to have to work at their relationship. As Kay Eagles explained.

‘…*not giving up… you have to work at relationships, they don't just happen… people change as they get older and relationships change, and the nature of relationships change all the time… the… falling in love bit is very much just… the glossy part at the beginning but doesn't necessarily give you the skills for a long, healthy relationship’.*

Fun and friendship were viewed as a necessity by many relationship professionals, not least because this gave a bedrock to come back to if couples begin to drift apart. Alongside this need to prioritise the relationship was a recognition of the need to maintain a sense of self. One of the relationship professionals described this concern for the self and the other as ‘*like a dance’*. As Jacob Beardsley put it, what is critical is.

*’the importance of looking after yourself in a relationship, thinking about yourself as a separate person as well as nourishing and caring for the relationship’.*

The relationship professionals distinguished the skills needed to initiate a healthy relationship and those needed to sustain it. The former included having: realistic expectations, a sense of self-worth, sufficient self-awareness to judge compatibility, well-developed communication skills and an ability to give and receive support within the relationship. The latter also included good communication skills as well as empathy, flexibility, likeability, commitment, respect, altruism, reciprocity and, in particular, resilience.

#### Theme 2—learning to relate

As might be expected, the relationship professionals spoke at length about the importance of good early caregiving in building the capacities of YP to form and maintain healthy relationships of their own. Positive early care, usually from parents, and the witnessing of a healthy, well-functioning relationship between parents was described variously as ‘*the building blocks’* (Margot Hendon),’*the architecture’* (Clara Farley) or’*the template’* (Fran Clarkson) for YP to learn relationship skills. The relationship professionals emphasised that a poor beginning did not mean that YP were doomed to make poor relationship choices or find maintaining relationships impossible. For some, positive other role models, grandparents or a teacher, might.

* ‘mediate some of those original depravations’. (Alexander Ingle).*

For others, counselling (preferably at a young age) was said to help YP gain skills that are not being modelled in the home or help YP understand that their parents’ behaviour is unhealthy.

Whilst a minority thought there was a place for peer mentoring and learning from one's peers more generally, several relationship professionals expressed concerns at the calibre of the training given to peer mentoring, the misinformation that peers can impart and the potential lack of objectivity of one’s peers.

Several relationship professionals spoke of the changes that would be needed at a macro level to cultivate an environment that.

*‘enables, or even supports individuals to establish and nurture relationships’. (Margot Hendon).*

#### Theme 3 – teaching about relationships in schools

While young people’s families were seen as the primary source of learning about healthy relationships, there was clear support for schools’ role to augment this. Relationship professionals thought that there were key transition moments in life, getting married or having a baby, where people are receptive to learning relationship skills, but that schools had a critical role in teaching and embedding critical skills around initiating and maintaining a healthy relationship.

There was strong support for Relationship Education to start early, preferably in primary schools, exploring what a healthy friendship and relating well to others looks like before moving onto romantic relationships, which would give YP vital life skills. Starting early, in primary schools and with counselling support where needed, was thought to be particularly important for YP whose parents were locked in conflict.

’*Modules that stress good relating from the very beginning*…*Once you get [skills to relate well to others] in your fold, and once you have got your template for good relating, it doesn't matter whether it's love relationships or with your teacher or with your friends or with anyone*’. (Rosemary Allen)

’*it is harder to unpick some of those really entrenched beliefs around relationships and things the longer it goes on*’ (Shelley Jackson)

Regarding content for a Relationship Education curriculum, teaching skills such as relating, communication, empathy, respect, conflict resolution and repair and ending relationships kindly and safely were highlighted. There was general agreement that these skills were teachable and that YP needed opportunities to rehearse using these skills to help them to recognise, for example, key turning points in interactions which leads some to end positively and others not. Therefore, there was strong support from the relationship professionals for RSE to be interactive and participatory, giving YP the opportunity to learn and try out vital communication skills in RSE by practising listening and mirroring what is heard in a non-conflict discussion.

’*[engaging] with an interaction that they can see somebody else having and they can then have input into trying to understand why the interaction went in the direction it went and how it might have gone differently and had different endings is… powerful’*. (Margot Hendon)

Regarding delivery of the teaching, Clara Farley felt that if Relationship Education lessons were to take place within schools, they needed to be ’*brilliantly led’* with ’*vivid and interesting materials’*, but she felt that schools did not have such material available to them. Others expressed reservations at asking teachers who may be experiencing difficulties in their own relationship to be responsible for Relationship Education in school. Another favoured external specialists to deliver Relationship Education, which was suggested as having added benefits.

*’young people are more likely to explore things, open up and be honest with someone that they perhaps haven't seen before, might not see again or see now and again around school. They will be more likely to share and explore their own thoughts than if they were with their own form teacher doing those things’. (Shelly Jackson)*

The relationship professionals were also in agreement that the emphasis of Relationship Education should be on teaching about healthy relationships in an inclusive way, which assists those in relationships that may be unhealthy because it allows them to reflect on differences.

’*[Relationships] come in all different shapes and sizes and sexual orientation and everything else, but nevertheless I think that it is possible to talk about at least, explore what a healthy relationship looks like in its many different forms*’. (Jacob Beardsley)

Several relationship professionals spoke of the need for excellent pastoral care and counselling within schools for YP with particular issues around relationships. Kay Eagles felt that Relationship Education should not just be limited to intimate relationships but relationships more widely to include components of respect, valuing and caring for others. Echoing the views of others, Alexander Ingles stressed the need for the ethos of the school to complement the messages within Relationship Education which should encourage YP to ask questions, with support readily available within schools.

‘[Relationship Education] can only work if it's in the context of a good school in a broad sense’. (Alexander Ingles)

## Discussion

Four main themes were presented from our focus groups with YP. The first, ‘Good and bad relationships’, presents YP’s views on romantic relationships, and the influences they recognised from parents and culture. The second, ‘Learning about relationships’, explores participant’s views of the benefits of Relationship Education and the skills they want to develop.

The third theme, ‘the role of schools’, is about experiences of Relationship Education teaching in the school setting and how and when this should be taught. The final theme of ‘Beyond Relationship Education’ focuses on some of the limitations of teaching relationships, and YP’s needs for support beyond the classroom. From the interviews with relationship professionals, we identified three relevant themes: what they viewed as the essential qualities of healthy relationships; how YP learn to relate (primarily through observing the parental role model) and the role that Relationship Education in schools might have in teaching YP how to instigate and maintain a healthy relationship. Many of the views of YP and relationship professionals were similar, but there were areas of contrast and variations in emphasis. Below, we discuss some of the key findings, drawing out implications for public health and education policy and practice.

YP and relationship professionals clearly recognised the importance of building relational capability. Relationship professionals emphasised the need for a developmental approach, which viewed relationships as requiring work rather than a more fatalistic view that relationships are either ‘good’ or ‘bad’ and that their trajectory is determined accordingly. An emphasis in Relationship Education on managing expectations, stressing that ‘good’ relationships do not just happen, as the relationship professionals advocated, would possibly counteract the “Disneyfied” portrayal of relationships in the media that the YP recognised were unhelpful. While the relationship professionals stressed the need for flexibility, adaptability, commitment and resilience as critical to maintaining relationships over the life course, these skills were not at the forefront of the YP’s minds. In contrast, YP often presented dichotomous views of relationships, possibly reflecting similar dichotomies presented in discussions at school around sex education [21]. YP appeared to be attempting to categorise relationships, using this dichotomous framework as a starting point. However, they also expressed a need to be able to better assess the quality or direction of a relationship, in order to take action, such as ‘cutting off’ a bad relationship. This was something they viewed as being a skill they could learn in Relationship Education. In line with the evidence discussed in the introduction, YP themselves also perceived a clear link between relationships and mental health; some raised this in terms of the impact of relationship breakdown, but there was also recognition that early relationships could set up ‘unhealthy’ patterns of relating which could affect mental health later in life.

Both the YP and the relationship professionals felt that schools were an important setting for teaching and learning about relationships, particularly in terms of offering what was seen as an ‘unbiased’ perspective and a universal offer. However, many YP felt that the existing Relationship Education offer was too ‘clinical’ and were keen to focus more on relational aspects. This view has been previously expressed in other research with YP around sex education, and by Ofsted’s review of the curriculum [21, 27]. Relationship professionals in our study called for a nuanced approach to Relationship Education that is skills-based and reflective of YP’s complex lived experiences [28]. The YP discussed the importance of building first on a strong ‘relationship with self’, which could be fostered through Relationship Education. Indeed, research suggests that self-compassion is associated with healthier romantic relationships [29] and many evaluations of Relationship Education programmes also measure ‘self-esteem’ as an outcome [30–33]. This chimed with the views of the relationship professionals who emphasised that ‘concern for the self’ was a prerequisite of being able to show concern for others. Related to this, the concept of building on previous knowledge and revisiting and reflecting on content as in a spiral curriculum [34] was also favoured by YP. For YP, the timing of the introduction of content around romantic relationships was more contested, with concerns over introducing pressure and expectations versus the risks of failing to address beliefs and norms until it was too late. The relationship professionals’ preference was to introduce age appropriate Relationship Education in primary schools.

Despite seeing benefits to Relationship Education, YP also identified limits due to its complexities and subjectivities, and some questioned whether this was a role for schools. This links to a broader debate about what education is for [35]. Several relationship professionals and YP interviewed highlighted the merits of trained external providers of RE. This resonates with Pound, Langford and Campbell [21] who found that YP want experts to teach them about sex and suggest that teachers should be specially trained and become a distinct group from other teachers. However, the DfE does not address these issues in its guidance [16].

### Strengths and limitations

This study is the only research we are aware of that explores in tandem young people and relationship professionals’ perspectives on the ‘relationship’ aspects of RSE. The nature of our sample presents some limitations, as it is possible that the YP were the most articulate and had the strongest views on Relationship Education amongst their peers and the relationship professionals who chose to engage may have had particular perspectives on Relationship Education. In particular, schools may have acted as ‘gatekeepers’ in selecting YP with more positive views on Relationship Education, however, we observed a range of views and dissent from focus groups across all settings. The inclusion of community and youth group members from different backgrounds increases our confidence that we have been able to explore and present a range of perspectives; it is also clear that YP’s views were not homogenous, hence dissenting voices are reflected in the themes. It is unclear as to the effect of the online format of the later focus groups; inevitably discussions require a higher level of moderation and direction. However, participants appeared comfortable with the format, and online research with YP has been found to potentially enhance their autonomy and amplify marginalised voices [36, 37]. The interview and focus group questions did not seek to explore or privilege relationship education from the perspective of any particular sexuality or identity. Researchers setting the scene were clear with YP that we wanted to explore how relationship education worked for all YP. However, whilst one young person alluded to needing to avoid ‘cookie cutter’ ideas of relationships within relationship education, and many participants used gender-neutral language or examples, we acknowledge that this research may be seen to feed into a heteronormative discourse which should be challenged and explored further in future work.

A purposive sampling strategy was used to recruit the relationship professionals. As selection of such a sample is subjective, purposive sampling is most appropriate for the selection of small samples, as here. Although, a limitation is that equally qualified relationship professionals, not known to the researchers by reputation, may have made different observations. There was a high degree of consensus across the sample.

#### Implications for policy and practice

This research is supportive of many aspects of curriculum guidance on Relationship Education. However, YP specifically highlighted areas that were priorities for them but are not explicitly addressed in the DfE’s RSE core content framework, such as managing relationship breakdowns, learning coping skills, and managing relationships through life course transitions. To engage YP in meaningful development and reflection during Relationship Education, the curriculum should reflect the content and skills that are relevant to them. Previous research has noted YP’s desire to be involved in future programme design [20], there was also support from the relationship professionals for Relationship Education to be co-created with YP, echoing the calls in the ‘Young People’s Manifesto’ [15].

Interestingly, YP and relationship professionals also wanted more of a focus on skills rather than knowledge. Professionals discussed the importance of providing opportunities for YP to observe and rehearse skills during lessons; and of engaging resources to support such learning. However, a recent survey of schools in England by Ipsos MORI and the PSHE Association [38] discusses the barriers faced in delivery of consistent and high quality Relationship Education, including knowledge, training and resources. Schools reported bringing in third sector organisations to deliver sessions and drawing on resources and lesson plans developed by organisations such as the PSHE Association (https://www.pshe-association.org.uk/). Cole [28] found support for the view that there is a lack of teaching proficiency, knowledge and confidence in the delivery of Relationship Education and teachers themselves viewed it as a specialist topic ‘outside their remit’. Currently, the DfE leaves schools to choose their Relationship Education curriculum content to meet their pupil and community needs, but there is clearly a need for schools to be better supported to deliver a more consistent approach to Relationship Education. This should include appropriate access to specialist expertise and resources, and guidance on signposting YP to external sources of help as required. Relationship professionals in our interviews also highlighted that positive relationship behaviours can also be modelled and integrated throughout school curricula and reflected in a school’s ethos. This links with existing research on the importance and influence of different types of relationships in schools on children and YP’s well-being and mental health such as peer to peer relationships, and those between teachers and pupils [39].

### Implications for research

This study raises a range of questions for exploration in future research, including the most effective ways to teach relationship skills, the best way to develop age-appropriate content, and how to integrate ‘relational health’ into a child’s journey through the education system. Available research is predominantly focussed on programmes developed to improve sexual health or reduce violence and abuse. Recent reviews by the authors [19, 20] have found few programmes focussing on healthy relationships, with a limited evidence base. However, as above, surveys suggest that most English schools do not use formal ‘programmes’ in any case. One implication is that research efforts are best focussed on the co-development, evaluation and implementation of education resources which can be used flexibly and integrate into a health promoting curriculum. A range of stakeholders, should be involved in co-development and evaluation, including YP, teachers, governors, parents, and others in the wider community who support YP’s well-being, as well as relationship professionals such as counsellors and mediators. These stakeholders should reflect the diversity of young people’s sexualities and identities, to ensure that Relationship Education is inclusive and accessible, and does not perpetuate inequalities or marginalisation. Understanding their perspectives on content, delivery, barriers, facilitators and desired outcomes is also necessary to ensure that Relationship Education is acceptable and feasible.

## Conclusion

This paper presents the perspectives of YP and relationship professionals on healthy Relationship Education and how it can better meet the needs of YP. Throughout the lifecourse, health comes about through relationships. Therefore, Relationship Education should form part of any approach to healthy publics [40] and is perhaps even more relevant in the light of the COVID-19 pandemic and the consequent societal impacts. Relationship building and Relationship Education should therefore be an integral aspect of a health-promoting school’s approach.

## Data Availability

The data that support the findings of this study are available on request from the corresponding author [SBC]. The data are not publicly available due to them containing information that could compromise research participant privacy/consent.

## Abbreviations

YP: Young people
RSE: Relationship and Sex Education
DfE: Department of Education
PPI: Patient and Public Involvement
HeaRE: Healthy Relationship Education
HeaRT: Healthy Relationship Transitions
COVID-19: The name of the disease caused by the novel coronavirus SARS-CoV2

## Footnotes

1 Relate, OnePlusOne, Tavistock Relationships, and Marriage Care.

2 Children and Social Work Act 2017, s 34(3)(a).

3 Henceforth education about relationships for 5 to 16-year-olds will be referred to as RE. This could include RE or RSE lessons as well as other direct and indirect forms of relationship education, including assemblies and aspects of the core curriculum.
